# Resignation of Professor Warren

**Published:** 1847-04

**Authors:** 


### RESIGNATION OF PROFESSOR WARREN.

Professor Warren has resigned the chair which he held for forty years in the medical department of Harvard University. In retiring he delivered an address to his students, extracts of which are given in the Boston Medical and Surgical Journal. We make room for the following:

" A kind and cheerful spirit should form a part of the character of every physician, whether young or old. Those visited by disease desire a cheerful sympathy in the physician who attends them, because they are thus induced to believe that he takes such an interest in their welfare as will lead him to a careful investigation of their symptoms, and to a diligent application of the means which may remove them. They regard every movement and expression with the utmost watchfulness, and their hopes rise or sink in a degree corresponding with these appearances. Many patients have lost their lives by a discouraging prognostic, and many have revived by a cheerful assurance of recovery.

" The morals of a young physician are not less important than his manners. He who has at his control, through the unknown remedies he may employ, the lives of his fellow men and the happiness of their families, requires a sensitive conscience to conduct him through all the mysterious and dangerous paths of medical duty. This sensitiveness is indispensable to the preservation of that interest which may languish under the pressure of professional fatigue. Doubt may sometimes obscure the course of his treatment, but whenever the indication is clear, no occupation, no apprehension should for a moment prevent its due execution."

